# Negative self-appraisal: Personal reasons for dying as indicators of suicidality

**DOI:** 10.1371/journal.pone.0246341

**Published:** 2021-02-02

**Authors:** Julian Madsen, Keith M. Harris

**Affiliations:** 1 School of Psychology, Charles Sturt University, Wagga Wagga, NSW, Australia; 2 School of Psychology, University of Queensland, St Lucia, QLD, Australia; Karolinska Institutet, SWEDEN

## Abstract

Reasons for dying (RFD) are one of the most authentic factors illustrating the lived experience of suicidal individuals. However, the field has been criticized for inadequate evaluation of risk factors and suicidal symptoms, such as RFD, to develop more robust theoretical models and risk assessments. In this study, we aimed to critically examine RFD themes as predictors of suicidal symptoms to improve our understanding of the suicidal mind, test suicide theory validity and improve risk assessment. This cross-sectional mixed-method study included anonymous survey data (N = 713) with a subsample (n = 474; 77% female; age M = 31.48, SD = 13.53) who provided RFD. Participants were asked to write down five RFD (ranked 1^st^ to 5^th^ most important) and completed the Suicidal Affect-Behavior-Cognition Scale (SABCS). Thematic analysis revealed eight valid RFD themes—Negative Self-appraisal, Hopelessness, Desire to Escape, Escape Pain, Relationships, Loneliness, Financial Hardship, and Physical Health. Themes were quantified by rank and total endorsements of the theme. Hierarchical regression modelling, statistically controlling for demographics, showed all RFD themes, except Physical Health, were positive predictors of suicidality, accounting for 26% of variance in suicidal symptoms. Negative Self-appraisal was the strongest predictor. RFD differences were also found by gender, age and education. From these findings, we determined current suicide theories do not fully account for suicidal persons’ RFD. There is a pressing need for more critical review of current theories, as current theories only partially represent this key attribute of the suicidal mind, and none of the reviewed theories accurately reflected suicidal participants’ RFD. Clinical implications include integrating financial therapies into suicide prevention treatments and incorporating RFD into assessments and treatments. To aid research and risk assessment efforts, we propose a new RFD Index, with eight five-point response items.

## Introduction

Understanding the reasons for suicide and establishing best practices for the assessment, prevention and treatment of suicidal behaviors, is a global public health imperative [1]. Nevertheless, the field has suffered from not effectively integrating risk factors and robust theoretical models of suicide into clinical practice and research [2, 3]. The replication crisis in psychology has been noted [4], but the lack of critical studies testing replication of suicide-related findings and theories may be beyond a crisis for this critical field. This study sought to improve our understanding of the drivers of suicide through re-investigation of individuals’ reasons for dying (RFD) as possible indicators of suicide risk. Gaining better perspective on the most common question asked–why do suicide victims want to die?–is also a sound starting point for reviewing suicide theory.

### RFD and suicidality

While RFD research is limited, there is evidence supporting associations between specific RFD in the development and maintenance of suicidality [5, 6]. Jobes and Mann’s [5] pioneering study of 49 patients from two American university counselling centers found the RFD themes–General Desire to Escape, General Descriptors of Self and Relationships with Others, accounted for 78% of clients’ RFD. Additionally, Mann [7] found the majority of RFD pertained to Descriptors of Self and a Desire for Escape in a study of 188 university counselling center clients. Together, these RFD themes accounted for about two-thirds of participants’ total RFD. In a small study of psychiatric inpatients, Grohmann [8] found Descriptors of Self, a Desire to Escape and Relationships with Others accounted for about two-thirds of RFD. While these studies identified common RFD of at-risk individuals, they did not demonstrate whether specific RFD are associated with suicidal symptoms.

In the largest study to-date (*N* = 1016), Harris and colleagues [6] found Hopelessness, Escape Pain, Descriptors of Self, Loneliness and a Desire to Escape accounted for about 80% of RFD in high-suicidal participants and that their RFD themes differed markedly from nonsuicidal participants. That study introduced a new RFD theme–None, referring to the absence of RFD. Later findings from Jennings [9], who examined an archival dataset featuring 120 adult suicide attempters, found Descriptors of Self, Relationships with Others and Hopelessness accounted for three-quarters of RFD. Across studies, Hopelessness, Desire to Escape, Relationships with Others and Descriptors of the Self, appear most salient. This suggests that negative self-focus and relationship difficulties are strong risk factors for elevated suicide risk. However, these findings require further examination and validation.

Much of the RFD literature features relatively homogeneous samples, including those low [5] [7] or very high in suicidality [8, 9]. Most studies, relied on specific cohorts, including military [10], clinical [8, 11, 12] and student populations [5, 7], with small sample sizes. There is a clear need to critically test the validity of these RFD themes and their relevance to suicide risk.

A further concern of much suicide literature regards the lack of theory to guide research and clinical practice [13, 14], which has stymied efforts to improve our understanding of suicide [15]. Theories can help explain why suicidal symptoms occur and might improve accurate assessment of suicide risk. While the theoretical literature has grown in recent years, no dominant theory has emerged in the field [3] or sought to specifically address the RFD of suicidal individuals. Theory is important for several reasons, including spurring further inquiry and to improve prediction. However, while theory is important, theories also need to be critically tested and their validity confirmed through repeated empirical evidence.

### Study aims

This study sought to examine community members’, across the suicidal spectrum, on their self-described RFD as potential predictors of suicidality. To improve generalizability, we sought to statistically control for potential demographic factors (gender, age, education, rurality and ethnicity). In addition, we critically reviewed popular suicide theories in relation to their inclusion of validated RFD themes. As a primary aim of this study was to critically re-evaluate the validity of previously identified RFD themes, with the possibility of identifying new themes, we made no specific hypotheses regarding which RFD might prove to be most relevant to predicting suicide risk.

## Method

### Participants

Ethics clearance was obtained by the host ethics review committee (H-2019-191590). The study represents a reanalysis of a previously reported cross-sectional study [16] using unanalyzed RFD data. Anonymous online survey participants (*N* = 713) provided responses to the questionnaire. However, this study focuses on a subsample (*n* = 474) who provided at least one RFD response. They were aged 18–71 years (*M* = 31.48, *SD* = 13.53); 77.1% were women; 68% identified as Caucasian/white and 31.8% as various other ethnicities; 55.8% were from Australia or New Zealand, 23.5% from other English-speaking countries; 42.2% lived in urban areas, 36.3% in towns, and 21.5% in rural areas.

### Measures

#### RFD and demographic items

Participants were asked to write down and rank, by importance, up to five RFD (ranked 1^st^ to 5th). There were no word restrictions. These items are consistent with the Suicide Status Form [17]. The SSF is central to the collaborative assessment and management of suicidality (CAMS), which is a suicide-specific clinical intervention and treatment that has been effective in randomized controlled trials [18, 19]. The prompt asked participants to list “your reasons for wanting to die, in order of importance (1 = most important, 5 = 5th most important).” The study included demographic variables (e.g., gender, age).

#### Suicidal Affect-Behavior-Cognition Scale (SABCS)

The SABCS [20] included six self-report items that capture suicidal symptoms, which load strongly on a single factor. An example item is “Right now, how much do you wish to die?” The wish to live item was reverse-scored. These items were anchored “not at all” to “very much,” however, other items use various response options. The items were combined, with higher scores indicating greater suicidal symptoms (range = 5–44). The SABCS was developed through classical test theory and item response theory methods and demonstrated stronger unidimensionality and psychometric properties than comparable scales. In addition, the SABCS demonstrated an absence of differential item functioning, indicating that it measures suicidal symptoms comparably across gender, ethnic, age and urban/rural groupings. The SABCS has been recommended by the Emergency Nurses Association [21] and the ENIGMA Major Depressive Disorder Working Group [22]. For this study, internal consistency was high, coefficient α = .93, McDonald’s ω_t_ = .95.

### Procedure

This was a purposive survey, hosted at a university web address, with oversampling of suicide-risk individuals to better examine variable associations. It was promoted as a survey on Personality, Suicidality & the Internet, for anonymous volunteer participants. To ensure strict anonymity, no IP addresses or other identifying information were collected. Participants were recruited through Google and Facebook advertisements and snowballing. After confirming consent to participate and participants’ age (18+ years), they were asked to complete non-compulsory questions. When participants ended the survey, they were presented with the exit page where they were shown links and phone numbers to free and anonymous online support and were encouraged to use the support if they felt distressed. The survey took approximately 15–25 minutes to complete. An online survey was chosen because it was well suited to measure psychological constructs in stigmatized groups and difficult-to-reach populations [23].

### Analyses

This mixed-methods study first applied a qualitative template analysis to identify and code RFD themes. The dataset was cleansed, treating univariate and multivariate outliers, and replacing missing values through recommended expectation maximization [24]. Template analysis affords a clear, systematic yet flexible approach to data analysis [25]. Two trained raters first independently coded 50% of the data using an initial template based on a priori themes [5, 6, 17]. Inter-rater reliability was adequate, Cohen’s kappa = .77, indicating substantial agreement [26]. Further modifications to the template were found necessary to incorporate new themes (i.e., Financial Hardship and Physical Health), and to modify previously identified themes (e.g., Escape Responsibility, Escape General). Next, the final template was used to code the full dataset. All codes were reviewed by three researchers, and any disagreements were finalized through discussion and consensus.

Previous studies only analyzed the first order RFD, here, we developed a scoring system to help us account for all RFD statements, therefore providing a more robust analysis. We weighted RFD responses as follows: first-order RFD = 5, second-order = 4, third-order = 3, fourth-order = 2, fifth-order = 1. Incomplete responses, or participants indicating they had no RFD, were scored 0. This resulted in eight RFD categories with scores ranging 0–15, however, as participants could endorse a maximum of five themes, theme scores were not well distributed across the full range. To reduce distribution issues, z-scores were created to inform collapsing categories. Themes were then rescored on a 7-point range. We produced a hierarchical regression model to test our primary research question of which specific RFD themes are most relevant to suicidal symptoms. We calculated squared semi-partial correlations (*sr*^*2*^) to identify the unique variance in the outcome variable (suicidality) explained by each predictor, after controlling for the effects of all other predictors. No influential outliers were discovered by Mahalanobis distance scores, regression statistical assumptions were met, and a post hoc power analysis indicated that sample size exceeded requirements for the proposed analyses (*N* > 107). To better generalize results to a broader population, we statistically controlled for relevant demographic factors, report adjusted *R*^*2*^ values and bootstrapped CIs for regression modelling [27, 28]. Analyses were done in SPSS v.26 (IBM Corp.) and R v.3.6.2. (R Core Team, 2019).

## Results

**Fig 1:**
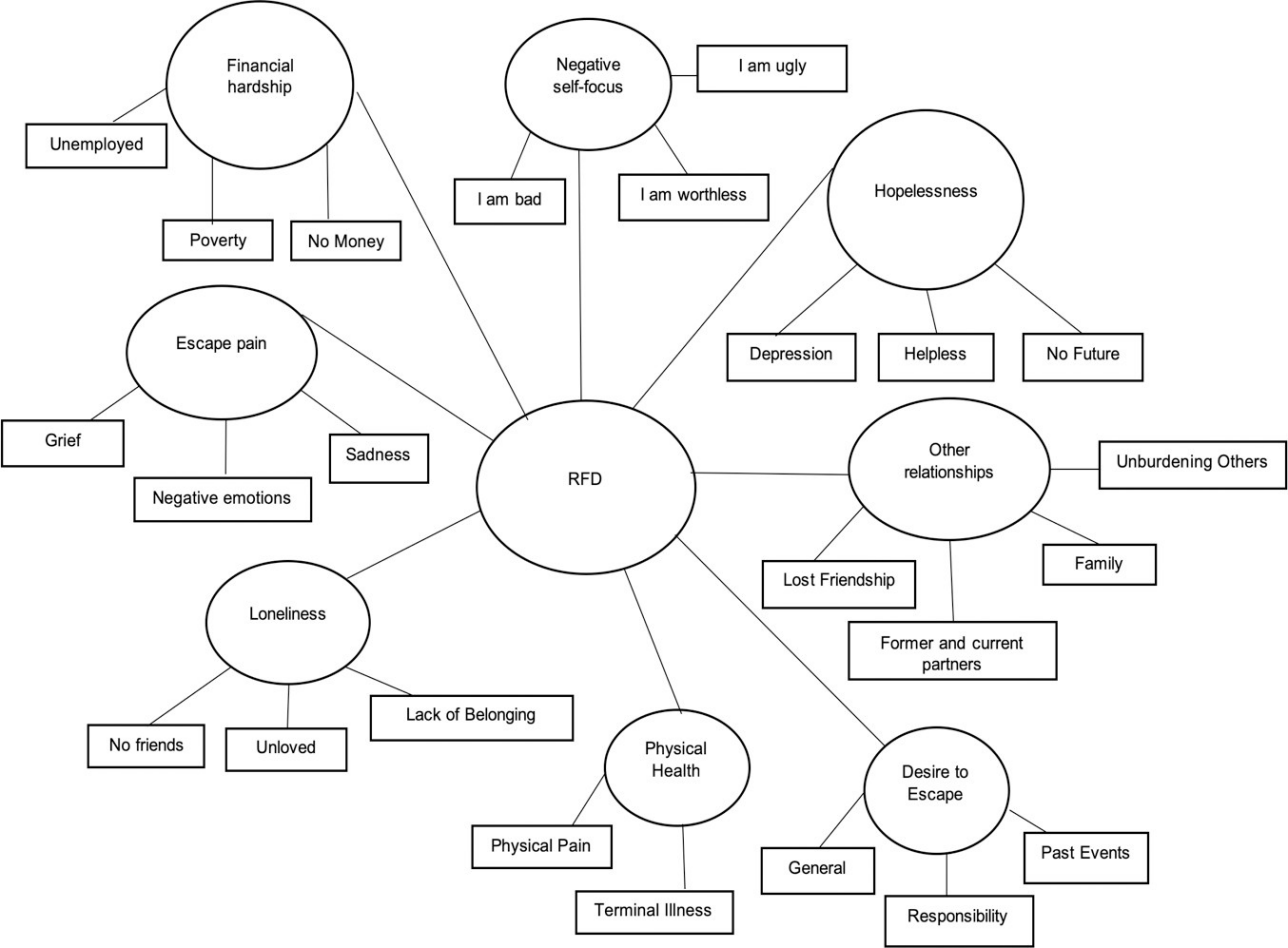
Reasons for dying themes.

Template analysis included 474 participants who provided at least one RFD. As our goal was to first define reasons for dying themes, we excluded responses where participants indicated no RFD, such as “no,” “none,” or “I don’t want to die.” We first compared participants who provided valid RFD with those who did not (*n* = 234). Chi-square tests showed no statistically significant group differences on gender, ethnicity, or regional residence, *p*s > .05. Point-biserial correlations showed the two groups statistically differed by small effect sizes with RFD reporters less educated (*r* = -.15, *p* < .001) and younger (*r* = -.08, *p* = .035). The groups showed a larger difference on SABCS scores, with RFD responders reporting higher suicidality (*r* = .44, *p* < .001). We examined SABCS score ranges by group, finding both subsamples included the full range (5–44), however, the non-response group had no cases for some higher range SABCS scores, while the RFD group fully covered the suicidal spectrum–as measured by the SABCS. The RFD responses were brief, most 1–7 words, producing 1,996 data-driven codes. Eight RFD themes were identified in the final template analysis (Fig 1) and are described below.

### RFD themes

#### Negative self-appraisal

This theme represents references to and feelings about the self and their personal beliefs and values. These responses were diverse, but statements involving negative self-worth were most salient. Examples included “[I’m] worthless,” “I’m a piece of crap” and “I don’t deserve to live.” Seeking to avoid failure and concerns regarding the future were also salient, such as “insecurity over my future.” There were responses about unaccomplished goals and current state of mind such as “disappointment in myself” and “not being successful.” Negative interpersonal relationship appraisal was also present, including “I’m socially awkward” and “everyone hates me.”

#### Hopelessness

This theme featured statements about hopelessness, including references to “depression,” feelings of “hopelessness,” having “nothing to live for” and their lives being “meaningless.” Many responses suggested that participants felt stuck “trapped in a futile life,” life being “too hard” and a “constant battle.”

#### Escape pain

This theme featured statements about psychological pain, including references to anxiety, grief and sadness, as well as negative emotions, such as anger and shame. Direct references to pain included statements about “make [the] pain stop” and “never-ending pain” and references to bereavement, such as “my brother passed away” and “my son committed suicide.” Being anxious and facing stressful situations, both vague and work or education-related, were salient and included being bullied at work and personal relationships.

#### Desire to escape

This theme featured statements about escape, such as references to the past or getting away from past experiences and feelings, getting out of responsibilities and general attitudes of giving up. There were diverse references to being “tired,” “feeling exhausted,” wanting to “rest” and “escape”. Desire to escape responsibilities and expectations were also salient and included statements about “major responsibilities,” “work,” “school,” “pressure to do well” and “expectations.”

#### Relationships

This theme included explicit and inferred references to other people. Statements about difficult relationships with identified individuals were varied, including with parents, partners, ex-partners, children, friends and pets were present and included references to “problems” and “issues.” A few statements referred to participants seeking to avoid burdening or hurting others, such as “becoming a burden” and “I’m just a burden to everyone around me.”

#### Loneliness

Statements about being “lonely,” “alone” and suffering from “loneliness” were highly salient. Other references to unmet social needs centered around love. For example, participants expressed feeling “unloved” and “nobody cares for me.” Not belonging and the lack of social connections were present through statements such as “no friends,” “not belonging,” being “rejected” and “abandoned.”

#### Financial hardship

This theme addressed financial difficulties specific to the individual, explicit or inferred. References to money, including having “no money” and “money worries” were salient as were statements about work and (lack of) employment, such as “jobless,” and “no work.” References to financial difficulties included statements about “financial stress,” “financial problems” and “financial debt.”

#### Physical health

This theme addressed references to serious health problems, either present or future. Direct statements to “ill health” and being “sick” were present. References to physical pain included “pain in my knees,” and “life-limiting pain.” There were also statements about specific illnesses such as “brain cancer,” being a “paraplegic” and contracting “HIV.” Some responses suggested these were hypothetical RFD based on the presence of ‘if’ before the statement, such as “if I was terminally ill” and “if could not function due to medical condition.”

**Fig 2:**
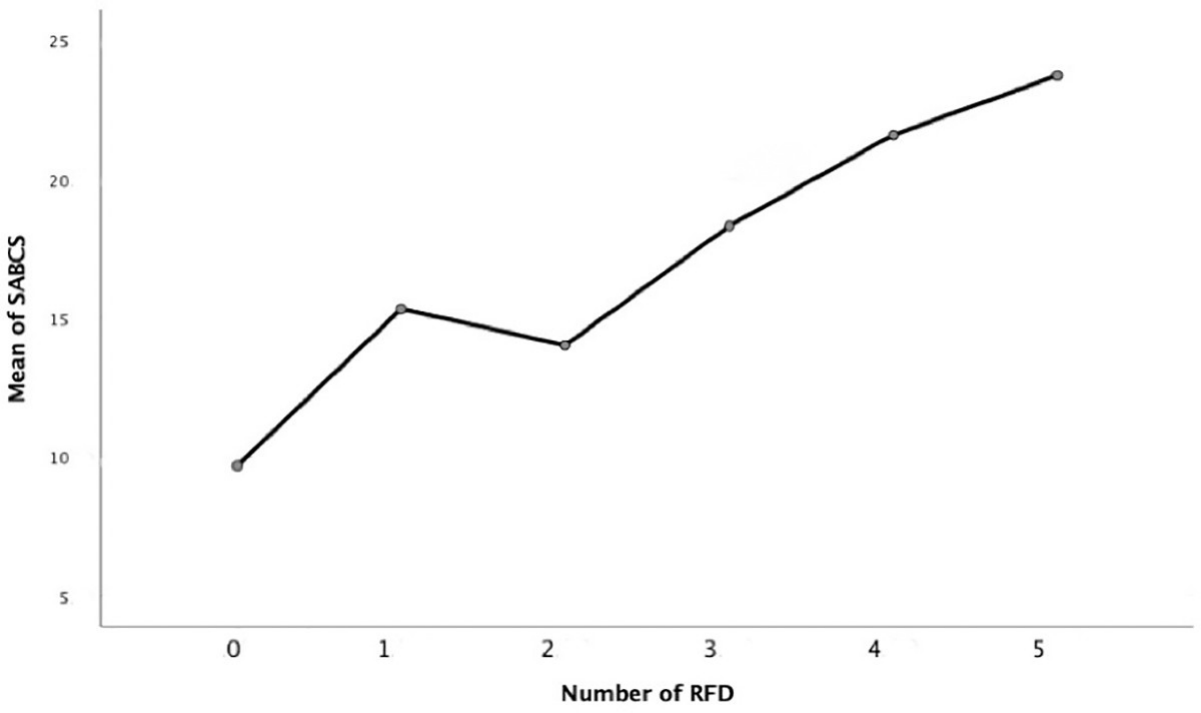
Association between the number of reasons for dying (RFD) and suicidality (SABCS scores).

We then explored the significance of how many RFD (range 0–5) participants indicated in relation to suicidality scores (SABCS). Fig 2 illustrates the nearly linear relationship between RFD count and suicidality symptoms. These data show that participants with minimal symptoms were unlikely to indicate any RFD. There was no apparent difference between those indicating one or two RFD, but 3–5 RFD showed a strong positive linear trend. These results provided some validation for quantifying endorsements of RFD by totaling the number of times each RFD theme was referenced.

Frequencies of completed RFD responses and rank order are presented in Table 1. Negative Self-appraisal and Hopelessness as most salient, endorsed at least once by more than half of participants who provided RFD. Financial Hardship and Physical Health were least common, but each were endorsed by over 20% of participants.

**Table 1:** Frequency of responses by ranked order reason for dying themes (n = 474).

RFD	1st	2nd	3rd	4th	5th	Total	Percent
Negative Self-appraisal	76	88	65	66	53	348	18.9%
Hopelessness	90	67	41	61	59	318	17.3%
Escape Pain	79	69	60	45	33	286	15.5%
Desire to Escape	76	61	50	34	42	263	14.3%
Relationships	63	53	57	45	28	246	13.4%
Loneliness	34	26	41	30	33	164	8.9%
Financial Hardship	19	32	27	21	9	108	5.9%
Physical Health	37	23	23	13	12	108	5.9%

### RFD and demographics

We next tested RFD themes by demographic factors and prediction of suicidality. For these analyses we used the complete sample, with non-RFD responders coded as ‘no’ RFD. Correlations showed statistically significant but small associations between age and RFD themes including negative self-appraisal (*r* = –.18 *p* < .001), loneliness (*r* = –.11, *p* = .004), financial hardship (*r* = .10, *p* = .005) and physical health (*r* = .22, *p* < .001); and for education with negative self-appraisal (*r* = –.17 *p* < .001), and loneliness (*r* = –.08 *p* = .032). One-way ANOVAs tested for gender, ethnicity and regional differences by RFD themes. The only statistically significant difference showed females were more likely to indicate Escape Pain (*M =* 0.85, *SD* = 1.51) than males (*M =* 0.50, *SD =* 1.16), *F*(1, 711) = 7.35, η_p_^2^ = .01, *p* = .007; and Caucasian/European participants were also more likely to report Escape Pain, (*M =* 0.87, *SD* = 1.53) than other ethnicities (*M =* 0.42, *SD =* 1.04), *F*(1, 711) = 11.80, η_p_^2^ = .02, *p* < .001; while rural/remote participants were more likely than others to report Negative Self-appraisal, *F*(2, 710) = 3.64, η_p_^2^ = .01, *p* = .027. Due to these findings, we statistically controlled for age, gender, education, ethnicity, and regional residence in the following regression modelling.

### RFD and suicidality

We next conducted a hierarchical regression model, with demographic variables entered as Step 1, to examine the associations between endorsing specific RFD and suicidal symptoms. Table 2 shows demographic variables explained a statistically significant 9% of suicidality variance: *R*^*2*^_*adj*_ = .09, *F*(5, 707) = 14.44, *p* < .001. At Step 2, RFD variables explained an additional 26% of suicidality variance, Δ*R*^*2*^_*adj*_ = .26, *F*(8, 699) = 35.54, *p* < .001. Other than Physical Health, all RFD were statistically significant predictors of suicidality. Based on *sr*^*2*^ coefficients, Negative Self-appraisal stood out as the strongest predictor of suicidality.

**Table 2:** Central tendencies and hierarchical regression model of demographics and RFD predicting suicidality.

Predictors	*M*	*SD/SE*	95.0% CI	*β*	*sr*^*2*^	*p*
Step 1						
Male	0.23	0.42	[0.20, 0.26]	.03	.00	.441
Education	2.26	1.06	[2.18, 2.35]	–.19	.03	< .001
Rural residence	0.79	0.77	[0.74, 0.85]	.15	.02	< .001
Caucasian/European	0.79	0.41	[0.75, 0.81]	–.11	.01	.005
Age	31.48	13.53	[30.48, 32.52]	–.05	.00	.182
Constant	B = 23.95	1.68	[20.44, 27.14]			< .001
Step 2						
Education				–.12	.01	< .001
Rural residence				.11	.01	< .001
Caucasian/European				–.11	.01	.002
Male				.05	.00	.093
Age				.03	.00	.441
Constant	B = 13.29	1.57	[9.82, 16.68]			< .001
Reasons for dying						
Negative Self-appraisal	0.85	1.70	[0.74, 0.98]	.33	.10	< .001
Hopelessness	0.81	1.47	[0.72, 0.92]	.20	.04	< .001
Desire to Escape	0.71	1.44	[0.61, 0.81]	.18	.03	< .001
Relationships	0.65	1.35	[0.56, 0.75]	.16	.03	< .001
Loneliness	0.40	1.00	[0.33, 0.48]	.18	.03	< .001
Financial Hardship	0.28	0.92	[0.21, 0.35]	.14	.02	< .001
Escape Pain	0.77	1.45	[0.67, 0.88]	.08	.01	.012
Physical Health	0.31	1.02	[0.23, 0.39]	-.01	.00	.764

*Note*. RFD = reasons for dying. Suicidality = Suicidal Affect-Behavior-Cognition Scale scores, ethnicity coded White/Caucasian or other, rurality (0 = rural, 1 = town, 2 = city). B = regression coefficient, with standard error. CIs and *p* values bootstrapped 1000 iterations.

## Discussion

We conducted a thorough re-examination of reasons for dying in relation to thematic validity and associations with suicidal symptoms. This study was unique in evaluating up to five RFD per participant, rather than only first-order RFD analyzed in previous studies. RFD were then weighted by their reported rank/importance. Our critical analyses of this more extensive data helped restructure previous RFD themes. Overall, we found an individual’s RFD was strongly related to their suicide risk, as measured by a highly valid scale. RFD themes explained a meaningful 26% of suicidality scores, after accounting for demographic factors. We also found a strong association between the number of reported RFD and suicidality. As the number of individual RFD rose, so did suicidal symptoms, which is consistent with previous research [6]. Seven RFD themes were predictive of suicidality. However, Negative Self-appraisal stood out as the most salient RFD. In contrast, Physical Health RFD showed no meaningful association with suicidal symptoms.

A primary aim of the current study was to test the replicability and validity of RFD themes. This was achieved through careful but critical analysis of previous studies [5, 6] and a thorough review of the present large data set by multiple researchers. Knowing an individual’s RFD can be highly useful for clinicians and others to manage personal suicidal symptoms [17]. Further, knowing which specific RFD themes are generally most related to suicidal symptoms offers additional possibilities for assessing risk and understanding suicidality. Based on squared semi-partial correlations from hierarchical regression modelling, we found six RFD themes demonstrated moderate to strong abilities to capture unique variance of suicidal symptoms. In contrast, the Escape Pain RFD showed a weak positive association with suicidality, and the Physical Health RFD was not associated with suicidal symptoms. Physical Health RFD showed signs of being a false theme for some, as many non-suicidal participants appeared to present this as a hypothetical RFD. Based on these findings, we highlight the need for clinicians and researchers to carefully review RFD for individual validity, particularly when prefaced with “if.” However, for older individuals, Physical Health did appear to hold some relevance as a high-risk RFD, indicating a need for attending to generational or personal circumstance differences.

### Negative self-appraisal

Negative Self-appraisal deserves particular attention as it was the strongest RFD predictor of suicidality and represented a third of all reported RFD. This theme whilst similar to the General Descriptor of Self RFD in previous research [5], is more specific in how suicidal people refer to themselves. There is substantial evidence of an association between Negative Self-appraisal (i.e., internal thoughts and feelings, attributes, physical characteristics, social roles, past experiences and future goals) and suicidality [29]. Depressed and suicidal individuals often have irrational thoughts involving themes of self-depreciation [30] and low self-esteem [31]. Overall, these characteristics of Negative Self-appraisal broadly support cognitive understandings of depression and suicide [32]. In addition, our review of the evidence shows that General Descriptors of Self is too broad a category. Negative Self-appraisal is a more accurate depiction of this highly suicidal perspective.

### Remaining RFD themes

Our findings that Hopelessness, Desire to Escape, Escape Pain, and Relationships are important RFDs fits well with theory and past empirical evidence. There is extensive evidence of a meaningful association between hopelessness and suicidal risk [33–35]. Hopelessness is considered to be a primary contributing factor in the development of suicidal thinking and behaviors [36, 37]. Desire to Escape was a significant predictor of suicidality here and is anchored in Baumeister’s suicide as escape from self theory [30]. There is considerable support for the psychological need for escape in the RFD research [6, 7] and non-RFD literature [38, 39]. Conflict in Relationships and Loneliness were positively associated with suicidality. Past research has also found that poor interpersonal relationships with to be associated with suicidal symptoms [40]. The current findings were also consistent with research indicating psychache or mental pain is a core construct of suicide [41, 42]. Although Escape Pain was the least predictive RFD of suicidality here, previous examinations of suicide notes suggested that escaping pain was a primary reason for suicide and suicide attempts [43, 44].

The Financial Hardship theme also predicted suicidality, which was a novel RFD finding deserving further attention. Dating back to Durkheim [45], there have been many theories and studies indicating associations between economic conditions and suicidality [45–47]. Recent evidence suggests financial hardship is positively associated with poor mental health [48], which in turn increases suicide risk [49]. The literature on financially focused interventions is somewhat limited [50], with little or no training provided to clinicians [48]. Financial therapy can be applied to enhance cognitive therapies through modifying and changing cognitions as well as monetary interactions [51].

### RFD and demographics

RFD themes showed important associations with demographic factors, which has been largely untested in previous works. We found significant differences among RFD and education attainment, gender and age, which were novel findings of this work. Those who only completed high school indicated more Negative Self-appraisal and Desire to Escape RFD than those with a university degree. This corresponded with research that indicated an inverse association between educational attainment and depression [52]. Higher levels of education are associated with higher levels of income and socioeconomic status [53], which are associated with lower levels of suicide risk [54]. Gender comparisons showed women were more likely to choose Escape Pain than men. This result seems to support research suggesting women are generally more sensitive to pain than men [55, 56]. The widespread adult psychopathologies, including the majority of anxiety disorders and depression, which affect more women than men [57] may also explain this study’s findings. In addition, younger participants reported more Negative Self-appraisal, Desire to Escape, Escape Pain, Loneliness and Financial Hardship RFD, whereas older people more often selected Physical Health. Financial hardship may be explained by younger people having fewer financial resources and physical health because people are more affected by poor health as they grow older. The increased selection of loneliness among young people corresponded with evidence that loneliness and social isolation peak in young adulthood [58] due to a lack of close friendships and romantic relationships [59]. Younger people’s choice of negative self-appraisal and desire to escape may be explained by evidence of higher levels of depression among young adults than older people [60, 61]. Future research is recommended to better decipher these patterns and their relevance to prevention and treatment efforts.

### Testing suicide theories

To demonstrate validity, suicide theory should reflect the commonalities of individual suicidal experiences [62]. We examined five popular suicide theories on whether they incorporated the most salient RFD found in this and previous studies. We found that none of the theories we evaluated fully incorporated the synthesized voices of suicidal lived experiences reported here. However, these theories were not equal in their representation of validated RFD. Baumeister’s theory of suicide as escape from self [30] appeared best in terms of representing the RFD of suicidal participants. While evidence supporting this theory is limited [63], it was the only reviewed theory to capture RFD related to Desire to Escape. However, its weakness was a lack of attention to relationships and loneliness. The integrated motivational-volitional theory [64] embodied the second-most RFD. Negative Self-appraisal and Loneliness RFD are well represented in the theory, but not the Hopelessness RFD. The cognitive model of suicidal behavior [65] represented three RFD–Negative Self-appraisal, Hopelessness and Financial Hardship. The interpersonal psychological theory [66] also includes three validated RFD–Relationships, Loneliness and Hopelessness. However, consistent with other studies [67, 68], that theory’s perceived burdensomeness hypothesis was not supported. Shneidman’s cubic model of suicide [69] includes Escape Pain, and partially represents the RFD of Loneliness and Financial Hardship. Each of these theories demonstrated strengths and weaknesses. Overall, we conclude that no current suicide theory is adequate from an RFD perspective. If a theory does not represent common themes of suicidal individual’s RFD, the validity of that theory might be questioned. Alternative theoretical viewpoints may be useful here. For example, the suicidal barometer model (SBM) is specific to suicide risk assessment [70]. The SBM postulates that suicide risk can be volatile, like the weather, and therefore should be assessed from a personal and momentary perspective, with attention to the suicidal individual’s internal debate between reasons for living and reasons for dying. We also note that each of these theories comes from a Western perspective. Improving our understanding of these reasons or motivations may require more culturally diverse contributions to suicide theory.

### Strengths and limitations

This cross-sectional study was not suitable for testing causation, or the consequences of advocating specific RFD. The large sample was useful for exploring demographic differences in RFD which was novel, however, additional large and diverse samples are required to confirm such variations. For example, further evaluation of the Physical Health RFD may show greater relevance with older or physical ill samples. This study had advantages over many previous studies, such as a more valid measure of suicidality, a large sample covering the full spectrum of suicidal symptoms (as assessed by the SABCS), and a weighting method to make use of all reported RFD themes. Also, notable, participants provided their personal RFD, with a high level of validity for those themes. However, participants were not in a position to comment on all eight validated RFD themes.

### Future directions: An RFD index

To further test the validity of the eight RFD themes, future research should examine these themes as specific questions. We propose the Reasons for Dying Index (RFD-I). The prompt reads: “Currently, to what degree do the following reasons for dying apply to you?” Followed by each of the eight RFD themes:

1. **Myself** (my character, how worthy I am, my beliefs) [i.e., Negative Self-appraisal–not shown]
2. **Hope for the future** (things may not get better, I can’t get over my problems) [i.e., Hopelessness–not shown]
3. **Getting away from my problems** (escaping problems, escaping my feelings, thoughts, responsibilities) [i.e., Desire to Escape–not shown]
4. **Escape pain** (ending my: pain, anxiety, loss, sadness, anger, shame, guilt)
5. **Relationships** (problems with my: partner, family, friends; I’m a burden on others, I’m underappreciated by others)
6. **Loneliness** (no-one understands me, I have no one, tired of being alone)
7. **Financial hardships** (lack of money or job, difficulty paying bills, no home)
8. **Physical health** (physical health problems now, worry about future physical health)

Responses can be scored: “1 = not at all,” “5 = very much.” Midpoints (2, 3, 4) labeled by number or not at all [71]. The RFD-I would provide data from all participants on each theme, allowing for more extensive analyses. This data would help confirm the validity of specific RFD themes as indicating higher or moderate risk by demographic groupings, thus providing greater insight into the suicidal mind for theory development, research and assessment purposes, but in a simple and timely manner.

## Conclusion

This study provided compelling evidence that the types of reasons for dying and the number of reasons for dying can indicate differing degrees of suicidality. These validated RFD may be used to develop new warning signs of suicide risk and provide strong support for the CAMS clinical approach. Part of CAMS’ aims are to identify, address and reduce RFD [17]. This study included the voices of hundreds of individuals across the suicidal spectrum. Their personal responses support current treatment emphasis on negative self-appraisal, hopelessness, and interpersonal relationships. We also recommend adding desires to escape as a pertinent suicidal factor, a possible suicide warning sign which may indicate a need for timely treatment. Clinicians should also consider integrating approaches that specifically address financial hardship.

The RFD validated in this study provided a unique and important view into the suicidal mind, particularly as RFD factors can differ between suicidal and nonsuicidal people. Based on the body of evidence, we recommend testing the RFD-I for comprehensive suicide risk assessments and treatment efforts. We also recommend carefully choosing suicide theories and measures to guide research and understanding. RFD provide a window into the suicidal mind; more attention to this crucial aspect of the individual’s experience can be useful in healing those minds.
